# Curcumin Induces Ferroptosis in Follicular Thyroid Cancer by Upregulating HO-1 Expression

**DOI:** 10.1155/2023/6896790

**Published:** 2023-01-14

**Authors:** Huanjie Chen, Zhoufan Li, Jingchao Xu, Ning Zhang, Junzhu Chen, Guangzhi Wang, Yongfu Zhao

**Affiliations:** Department of General Surgery, The Second Hospital of Dalian Medical University, Dalian Liaoning Province, China

## Abstract

Follicular thyroid cancer (FTC) is a highly aggressive type of endocrine malignancy. It is necessary to investigate the mechanisms of tumorigenesis and therapeutic pathways in patients with FTC. Haem oxygenase-1 (HO-1) can regulate oxidative stress and the occurrence of tumors and diseases. In this study, we discovered that HO-1 was abnormally overexpressed in FTC compared with adjacent tissues. However, the HO-1 overexpression was demonstrated to decrease cell viability and to potentially activate the ferroptosis signalling pathway. Ferroptosis is a newly identified form of oxidative cell death and is currently being targeted as a new cancer treatment. Tumorigenesis is significantly inhibited by curcumin. The present study shows that curcumin inhibits the growth of FTC by increasing the HO-1 expression, further activating the ferroptosis pathway. This study demonstrates that the HO-1-ferroptosis signalling pathway might play an important role in FTC tumorigenesis, and that curcumin inhibits the growth of FTC cells by affecting this pathway.

## 1. Introduction

Thyroid cancer is the most common endocrine malignancy and the fifth most common cancer among women in the United States, and it ranks fifth among new cancer cases worldwide [1, 2]. Thyroid cancer can be classified into four pathological types, among which follicular thyroid carcinoma (FTC) has the second highest morbidity rate, following papillary thyroid carcinoma (PTC). FTC is more aggressive than PTC and readily undergoes haematogenous metastasis. In addition, FTC is more difficult to diagnose in the absence of paraffin pathological sections to examine. In addition, it is difficult to distinguish between follicular thyroid adenoma (FTA) and FTC in thyroid nodule diagnosis [3]. To avoid missing FTC diagnosis, many FTAs have been surgically removed, leading to overtreatment [4]. Once FTC cells metastasize to distant organs, such as bone, brain, and lung, the mortality rate obviously increases, and surgical treatment can be ineffective or detrimental [5]. Thus, it is necessary to search for therapeutic interventions for FTC.

Haem oxygenase-1 (HO-1), a rate-limiting enzyme for haem degradation, can metabolize haem into biliverdin/bilirubin, carbon monoxide, and ferrous iron [6]. Multiple human malignancies are associated with elevated HO-1 levels, which contribute to the formation of a tumor microenvironment that supports cancer cell growth, angiogenesis, and metastasis. However, the increased HO-1 expression can promote cell death in many types of cancers [7–9]. Ferroptosis has recently been shown to differ morphologically, genetically, and mechanistically from necrosis, autophagy, and apoptosis [10]. Two features of ferroptosis are the inhibition of glutathione peroxidase 4 (GPX4) and the subsequent accumulation of iron-dependent lipid reactive oxygen species (ROS) [11]. Emerging evidence shows that HO-1 induces ferroptosis through iron accumulation [12, 13]. Moreover, the iron dependence of cancer cells makes them more prone than normal cells to ferroptosis [14]. However, the mechanisms by which HO-1 regulates ferroptosis in FTC remain unclear.

Research has demonstrated that curcumin has potential applications in pharmacology and cancer treatment [15]. Curcumin inhibits cancer cell growth and promotes apoptosis in colorectal cancer [16], lung cancer [17], and prostate cancer [18]. Additionally, numerous studies have shown that curcumin elevates the HO-1 expression in a wide range of cancer cells, and the upregulation of HO-1, which can degrade haem and synthesize ferritin, alters the way iron is distributed in cells [19]. Ferroptosis can be induced by the increased HO-1 expression through iron accumulation and ROS production [20], which means that curcumin and ferroptosis are closely related to each other because of curcumin's effects on HO-1. However, the mechanism by which curcumin inhibits FTC cells is unclear.

This study was designed to elucidate the molecular mechanism underlying the inhibitory effect of curcumin on FTC and to propose a new intervention for FTC. The results show that HO-1 is upregulated in FTC tumorigenesis. In addition, drug intervention experiments demonstrated that FTC tumorigenesis is inhibited by curcumin *via* a mechanism that may involve the direct action of HO-1 and the ferroptosis signalling pathway.

## 2. Materials and Methods

### 2.1. Sample Collection and Ethics Statement

FTC samples and adjacent tissues were collected and immediately frozen in liquid nitrogen. All of the samples were collected with the written informed consent of all individual participants in this research, and all of the FTC samples were confirmed as FTC by pathologists through histopathological evaluation. The research was performed in accordance with the Declaration of Helsinki. The study was authorized by the Ethics Committee of the Second Hospital of Dalian Medical University.

### 2.2. Cell Culture

Nthy-ori-3-1 (human normal thyroid cells), FTC-133, and FTC-238 (two types of FTC cells with different levels of invasiveness) cells were acquired from the Chinese Academy of Sciences' cell bank (Shanghai, China) along with Cell Line STR Authentication Reports. The Nthy-ori-3-1 cells were cultivated in RPMI 1640 with 10% foetal bovine serum (FBS). FTC-133 and FTC-238 cells were cultivated in DMEM with 10% FBS. All cells were cultivated in 37°C and 5% CO_2_ environments in incubators.

### 2.3. Next-Generation Sequencing

Three pairs of samples, collected from the Second Hospital of Dalian Medical University, were sent to Novogene (Tianjin, China) for next-generation sequencing.

### 2.4. Chemicals and Reagents

Erastin (M2679), ferrostatin-1 (Fer-1, M2698), and deferoxamine (DFO, M5129) were all purchased from AbMole Bioscience Inc. (Texas, USA). Curcumin (Cur, C7727) and autophagy inhibitor (chloroquine, CQ, C6628; 3MA, M9281) were purchased from Sigma (St. Louis, USA). Apoptosis inhibitor (Z-VAD-FMK, C1168 M-2) was purchased from Beyotime (Shanghai, China).

### 2.5. Immunohistochemistry

Human thyroid tumor specimens were acquired from the Department of Pathology. Tumor sections (3 *μ*m) were incubated with the primary antibody anti-HO-1 (1 : 100, Proteintech [10701-1-AP], USA) overnight at 4°C. The next day, the sections were incubated with goat anti-rabbit Envision System Plus-HRP for 30 min at room temperature. The sections were rinsed three times using phosphate-buffered saline (PBS) for 10 min each, and then diaminobenzidine (DAB) was used to incubate the sections for 2 min. The tumor sections were dehydrated and mounted after counterstaining with Mayer's haematoxylin. Finally, the sections were visualized by an Olympus microscope at a magnification of ×20. Two experienced pathologists examined the sections under a double-blind protocol. The results of immunohistochemistry were scored according to the percentage of positive cells and the intensity of cell staining. The scores for the percentage of positive cells were zero (no positive cells), one (≤25%), two (25–50%), three (51–75%), and four (≥75%), and those for the intensity of the cell staining were zero (negative for staining), one (weakly positive staining), two (moderately positive staining), or three (strongly positive staining). The final score for each specimen was the product of the two scores. Based on the arithmetic mean of these scores, the specimens scoring 8 or higher were deemed to have high expression.

### 2.6. Western Blotting Assay

Western blotting assays were used to detect the quantitative levels of protein expression. First, thyroid cancer cells or solid tumor tissues were lysed using radioimmunoprecipitation assay (RIPA) lysis buffer. After resolving the proteins on 10% SDS-PAGE gels, the proteins were transferred onto PVDF membranes, which were incubated with the primary anti-HO-1 antibody (1 : 1000, Proteintech, USA) or anti-GPX4 antibody (1 : 500, Proteintech, USA) at 4°C overnight. Next, the PVDF membranes were incubated with peroxidase-conjugated secondary antibody (1 : 3000, Proteintech, USA) for 2 h at room temperature. Western blotting results were calculated by using ImageJ software and normalized to the *β*-actin value.

### 2.7. Cell Viability Assay

To evaluate cell viability, cells were digested by trypsin at the logarithmic growth phase and evenly added to a 96-well plate (density: 5 × 10^3^ cells per well). Each group was represented by 6 replicate wells. The plate was placed in an incubator at 37°C in a 5% CO_2_ environment. The cells were allowed to adhere. The medium was then replaced with medium including the specified concentrations of reagents, and the cells were incubated for 24 h. Each well was then supplemented with 10 *μ*l Cell Counting Kit-8 (CCK-8; Meilun, Dalian, China) solution, and then the 96-well plate was incubated at 37°C for 1 h. The absorbance at 450 nm was measured using a microplate reader (Thermo Fisher Scientific, Shanghai, China).

### 2.8. Colony Formation Assay

A total of 1000 cells were placed in a 6-well plate and then incubated at 37°C in a 5% CO_2_ environment. After 1 week, the medium including the specified concentrations of curcumin was discarded. PBS was used to gently wash the residual medium. Cells were stained with 0.2% dissolved crystal violet after fixation with methanol for 30 min. Then, the number of colonies containing more than 30 cells was counted.

### 2.9. Migration and Invasion Assays

Cells were transferred to the upper chamber of a transwell membrane filter (SPL Life Sciences Co., Ltd., South Korea) after resuspension in serum-free medium, and 600 *μ*l medium containing 10% FBS was added to the lower chamber. The cells were then cultivated at 37°C in a 5% CO_2_ environment for 24 h. Dissolved crystal violet (0.2%) was used to stain the cells after they were treated with methanol. Finally, the cells were photographed and counted in three random fields by using an Olympus microscope (Tokyo, Japan) at a magnification of ×20.

### 2.10. Measurement of Iron, Glutathione (GSH), and Malondialdehyde (MDA) Contents

Iron contents were detected by using an Intracellular Iron Colorimetric Assay Kit (E1042, Applygen Technology Inc. Beijing, China). Cells were collected with precooled PBS and then centrifuged at 12000 rpm for 10 min. The supernatant was collected for the following tests. GSH is an antioxidant, and MDA is an end product of lipid peroxidation. Both are markers of oxidative stress. A GSH assay kit (Microplate method, A006-2-1) and an MDA assay kit (TBA method, A003-1) were purchased from Nanjing Jiancheng Bioengineering Institute. Cell samples were treated as described in “iron content detection” above. All operations were executed according to the manufacturer's instructions [21, 22]. The iron contents were calculated by measuring the absorbance at 550 nm. Individual contents of GSH and MDA were read at 405 and 532 nm, respectively, by a microplate fluorometer.

### 2.11. Lipid ROS Assay

Lipid ROS, considered one of the most important markers of ferroptosis, was measured by using Liperfluo (#L248, Dojindo, Kumamoto, Japan). We seeded cells in 24-well plates, pretreated them with curcumin-containing medium for 24 h, and incubated them for 30 min in the dark with Liperfluo at a working concentration of 1 *μ*M. PBS was then used to wash the cells three times. Finally, a fluorescence microscope (Olympus, Tokyo, Japan) was used to acquire images at a magnification of ×40.

### 2.12. Cell Transfection

Small interfering RNAs (siRNAs) with sequences targeting HO-1 or GPX4 were constructed by GenePharma (Shanghai, China).  Table 1 presents the sequences of these siRNAs. To produce cells overexpressing HO-1, the coding sequence for HO-1 was inserted into the pcDNA3.1 vector (HO-1-OE). The transfection steps were conducted following the manufacturer's protocols. P3000 and Lipofectamine™-3000 (Invitrogen, USA) were used to transfect the cell lines.

### 2.13. Quantitative Real-Time PCR Analysis

Total RNA was extracted from cells using an RNA extraction kit (Accurate Biology, AG21017). Evo M-MLV RT Premix (Accurate Biology, AG11706) and SYBR Premix Ex TaqTM (Accurate Biology, AG11702) were used to prepare the samples for RT-qPCR, which was conducted on a Rotor-Gene Q instrument (QIAGEN, Germany). We calculated the relative expression levels of target genes based on the 2^-*ΔΔ*CT^ method.  Table 2 lists the primer sequences.

### 2.14. Coimmunoprecipitation (Co-IP)

According to the manufacturer's instructions (Proteintech, PK-10007), the interaction between HO-1 and GPX4 protein was evaluated by Co-IP. Protein was extracted from cells by using IP lysis buffer supplemented with protease inhibitor. A BCA assay was used to measure the protein concentrations. The HO-1 antibody or IgG antibody was mixed with protein extracts and then incubated overnight at 4°C with incubation buffer. The following day, antibody-conjugated beads were used to immunoprecipitate HO-1 and its interacting proteins for 2 h at room temperature. Washing buffer was used to wash the protein-bead complexes five times, and then the protein was eluted with 40 *μ*l elution buffer two times. Finally, western blotting analyses were performed on the precipitated proteins.

### 2.15. Statistical Analysis

GraphPad Prism software (GraphPad 8.0, Inc., USA) was utilized for all statistical analyses. The results are presented as the means and standard deviations (SDs) of three replicates per group. For comparisons between different groups, experimental data were analyzed using one-way/two-way analysis of variance (ANOVA) or *t* test. A chi-square test was used for immunohistochemistry analysis. *p* values less than 0.05 were considered statistically significant.

## 3. Results

### 3.1. HO-1 Expression Is Upregulated in FTC Tissues and Cell Lines

**Figure 1:**
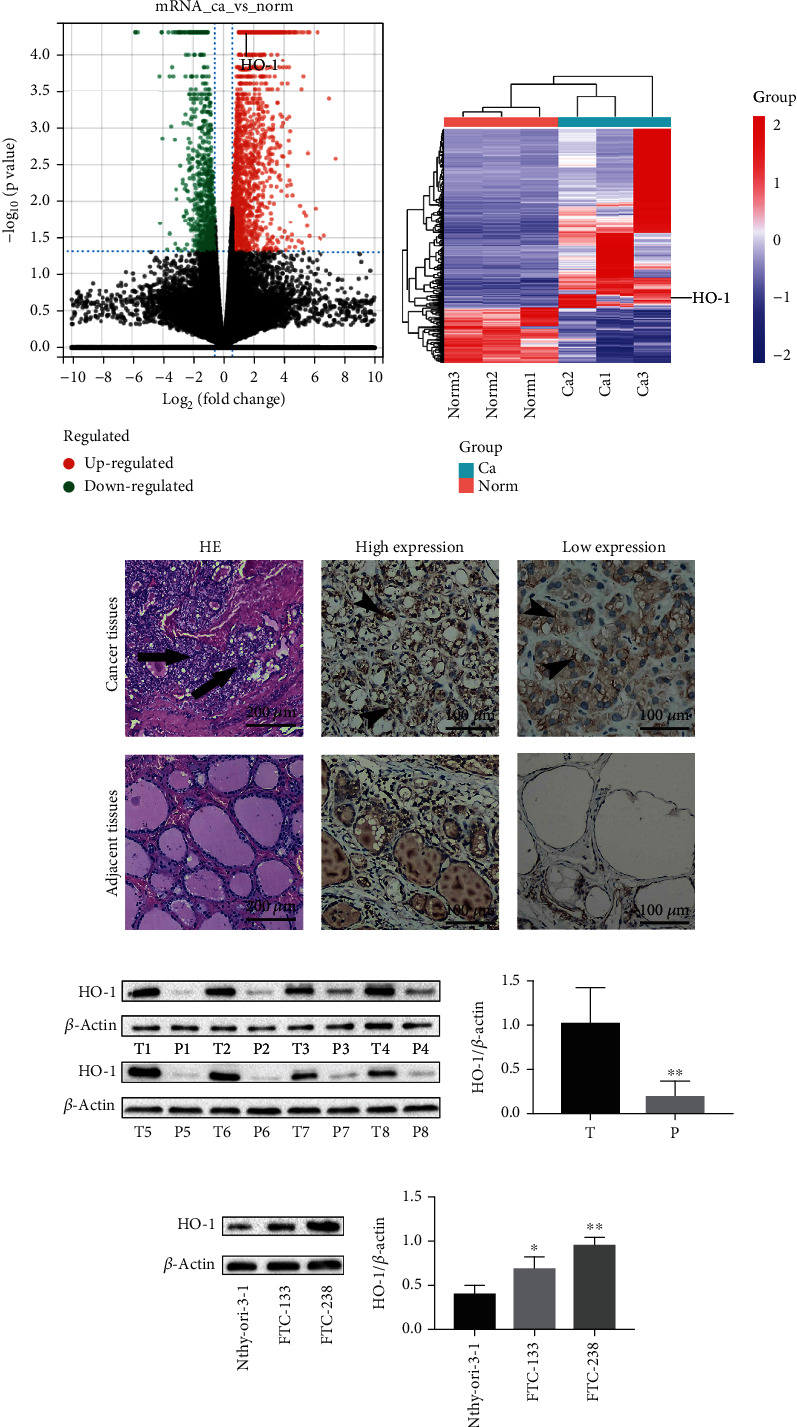
The expression of HO-1 is upregulated in FTC. Volcano plots (a) and heatmaps (b) show that the HO-1 expression is upregulated in FTC tissues compared to adjacent tissues. Ca: follicular thyroid cancer tissues; Norm: adjacent normal thyroid tissues. (c) Immunohistochemistry (IHC) results of the HO-1 expression in FTC tissues and adjacent tissues were analyzed by the *chi-square* test. The black arrows highlight locations where the tunica of tumor cells was invaded, as shown by hematoxylin and eosin (HE) staining. The black head arrows show the expression of HO-1 protein. HE, scale bars: 200 *μ*m; IHC, scale bars: 100 *μ*m. (d) The relative expression of HO-1 is shown as a percentage of the *β*-actin expression in FTC tissues and adjacent tissues (T: FTC tissues; P: adjacent tissues, *N* = 8). (e) HO-1 was overexpressed in FTC cells, particularly in FTC-238 cells, compared to Nthy-ori-3-1 cells. ^∗^*p* < 0.05, ^∗∗^*p* < 0.01. The data are presented as the mean ± standard error of the mean (SEM), *n* = 3. Statistical significance was determined by *t* test (d) and one-way ANOVA (e).

To investigate the role of HO-1 in the tumorigenesis of FTC, we conducted next-generation sequencing analysis and compared protein expression between cancer tissues and adjacent tissues from FTC patients. Significant differences were observed in the mRNA expression profiles. Volcano plots and heatmaps were generated to visualize expression differences, which were identified according to *p* < 0.05, *q* < 0.05, and the fold change **(**Figures 1(a) and 1(b)**)**. HO-1 mRNA levels were dramatically increased in FTC (fold change = 1.69587, *p* value =0.00005, and *q* value =0.00548452). Correspondingly, 33 pairs of thyroid cancer tissues and adjacent tissues were examined for the expression of HO-1 by using an immunohistochemistry assay (Figure 1(c)). The positive expression rates of HO-1 in cancer tissues and adjacent tissues were 61% and 33%, respectively (*χ*^2^ = 4.93, *p* = 0.026) (Table 3). The difference was statistically significant.

Similarly, a significant increase in the protein expression of HO-1 in cancer tissues compared to adjacent tissues was identified *via* western blotting assay (*N* = 8, Figure 1(d)**)**. In addition, western blotting assay was used to examine the HO-1 expression in three types of cells: Nthy-ori-3-1, FTC-133 (poorly invasive), and FTC-238 (highly invasive) cells. The results showed that the HO-1 expression was much higher in FTC-238 cells than in FTC-133 cells or Nthy-ori-3-1 cells **(**Figure 1(e)**)**. Accordingly, we hypothesized that the aberrant expression of HO-1 may be involved in the progression of FTC.

### 3.2. Effects of HO-1 on Erastin-Induced Ferroptosis in Thyroid Cells

**Figure 2:**
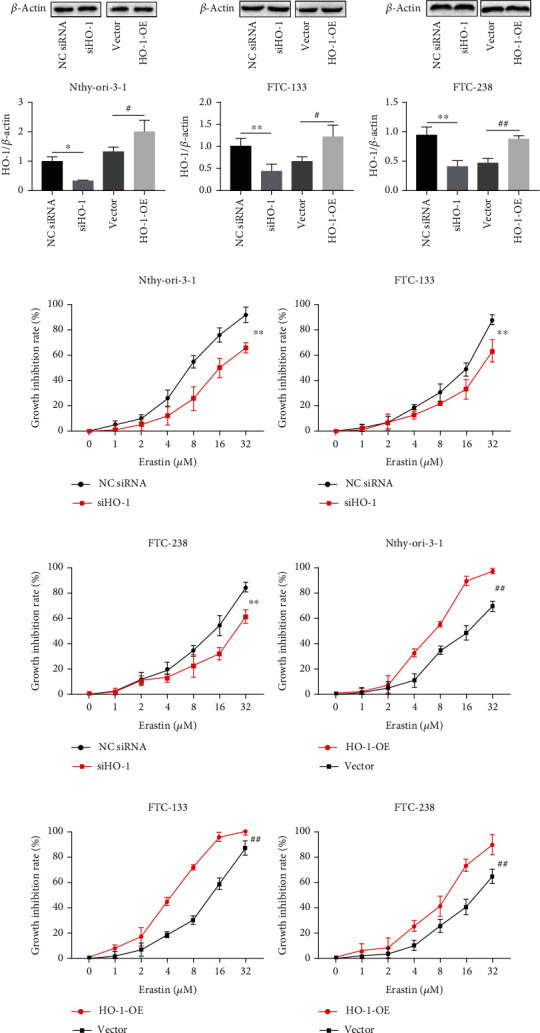
Transfection efficiency and growth inhibition rate. Transfection efficiencies of siHO-1 and HO-1-OE in Nthy-ori-3-1 and FTC cells (a–c). *β*-Actin was used as an internal control. (d–f) The growth inhibition rates of Nthy-ori-3-1 and FTC cells transfected with siHO-1 and treated with erastin (0, 1, 2, 4, 8, 16, and 32 *μ*M). (g–i) The growth inhibition rates of Nthy-ori-3-1 and FTC cells transfected with HO-1-OE and treated with erastin (0, 1, 2, 4, 8, 16, and 32 *μ*M). ^∗^*p* < 0.05 and ^∗∗^*p* < 0.01 compared with the Nc siRNA group. ^#^*p* < 0.05 and ^##^*p* < 0.01 compared with the vector group. The data are presented as the mean ± standard error of the mean (SEM), *n* = 3. The *t* test (a–c) and two-way ANOVA (d–i) were used to determine statistical significance.

Erastin, a classic ferroptosis inducer, can accelerate cell death through the accumulation of ROS [23]. To investigate whether HO-1 can affect erastin-induced cell death, we transfected Nthy-ori-3-1 and FTC cells with siHO-1 to knockdown HO-1. A recombinant HO-1-OE plasmid was transfected to overexpress HO-1. The transfection efficiency of each of siHO-1 and HO-1-OE was verified by western blotting assays. The protein expression of HO-1 showed obvious downregulation after transfection with siHO-1, and transfection with HO-1-OE significantly increased HO-1 protein levels (Figures 2(a)–2(c)). The CCK-8 assay results showed that siHO-1 inhibited erastin-induced death **(**Figures 2(d)–2(f)**)**. In contrast, HO-1-overexpressing Nthy-ori-3-1 and FTC cells were more sensitive than the corresponding siHO-1-treated cells to erastin-induced death **(**Figures 2(g)–2(i)**)**. These findings support the view that the high HO-1 expression may be related to the initiation of ferroptosis, especially in thyroid cells.

### 3.3. Curcumin Suppresses FTC Tumorigenesis

**Figure 3:**
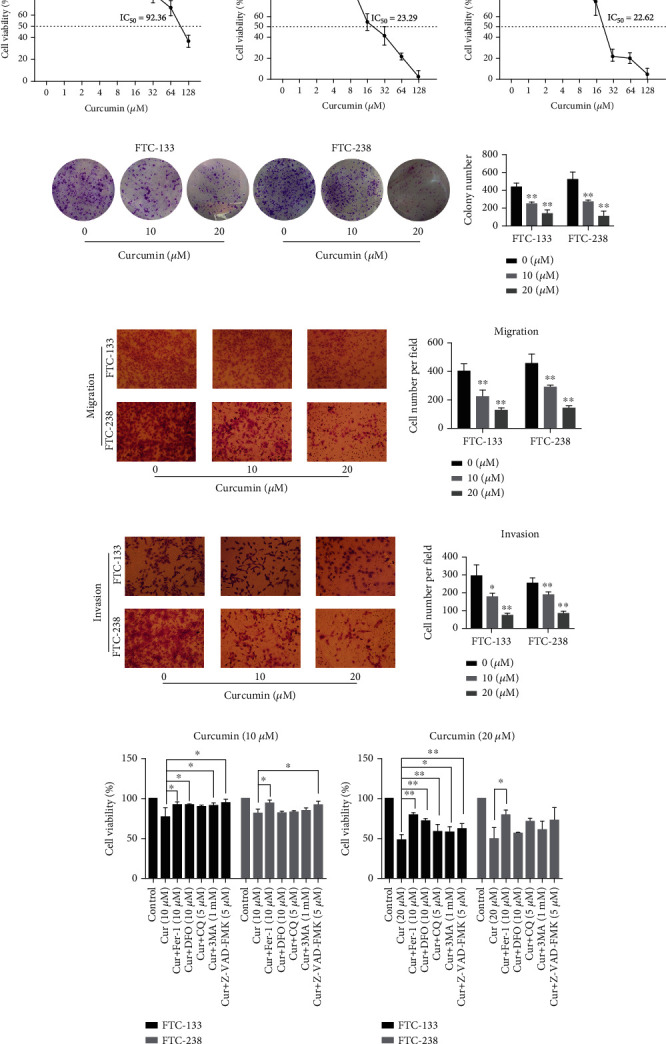
Curcumin inhibits FTC tumorigenesis. (a) Nthy-ori-3-1 and FTC cells were treated with the indicated concentrations of curcumin for 24 h, and curcumin inhibited cell viability. Cell viability was measured using CCK-8 assays. (b–d) Curcumin suppressed the growth of FTC cells. FTC cells were treated with 0, 10, or 20 *μ*M curcumin and subjected to colony formation, cell migration, and invasion assays. Scale bars: 100 *μ*m. ^∗^*p* < 0.05 and ^∗∗^*p* < 0.01 compared with the 0 *μ*M curcumin group. (e, f) FTC cells were either preincubated with various inhibitors, namely, ferrostatin-1 (Fer-1, 10 *μ*M), deferoxamine (DFO, 10 *μ*M), chloroquine (CQ, 5 *μ*M), autophagy inhibitor (3MA, 1 mM), and Z-VAD-FMK (5 *μ*M), for 2 h and then treated with 10 or 20 *μ*M curcumin (Cur) for 24 h or treated with curcumin alone. Cell viability was assessed using CCK-8 assays. ^∗^*p* < 0.05 and ^∗∗^*p* < 0.01 compared with the group receiving curcumin treatment alone. The data are presented as the mean ± standard error of the mean (SEM), *n* = 3. One-way ANOVA (b–d) and *t* test (e, f) were used to determine statistical significance.

Curcumin has anticancer effects in multiple types of solid tumors [24]. To better understand the relationship between curcumin and thyroid cells, Nthy-ori-3-1 and FTC cells were treated with gradient concentrations of curcumin for 24 h. CCK-8 assays showed that curcumin treatment clearly decreased cell viability in a dose-dependent manner (Figure 3(a)), although Nthy-ori-3-1 and FTC cells reacted differently to curcumin. Specifically, the 20% growth-inhibitory concentration (IC_20_) values for FTC-133 and FTC-238 cells were 9.35 *μ*M and 10.56 *μ*M, respectively. Correspondingly, the 50% growth-inhibitory concentration (IC_50_) values for FTC-133 and FTC-238 cells were 23.29 *μ*M and 22.62 *μ*M, respectively. However, the IC_20_ and IC_50_ values for Nthy-ori-3-1 cells were 32.25 *μ*M and 92.36 *μ*M, respectively. Of note, only 32, 64, and 128 *μ*M curcumin significantly inhibited the viability of Nthy-ori-3-1 cells. These findings indicated that the FTC and Nthy-ori-3-1 cells differed in their sensitivity to curcumin treatment, and that low concentrations of curcumin inhibited the viability of the FTC cells without affecting that of the normal thyroid cells.

To examine the effects of curcumin on FTC cells, we selected concentrations of 10 *μ*M and 20 *μ*M for follow-up tests. The results of the colony formation assays demonstrated that curcumin treatment dramatically reduced the number of colonies formed (Figure 3(b)) and inhibited cell proliferation. Transwell assays were conducted to explore the effects of curcumin on the migration and invasion in FTC cells. Cell migration ability was obviously decreased after treatment with gradient concentrations of curcumin (Figure 3(c)). Similarly, transwell assays using Matrigel demonstrated that curcumin inhibited the invasion of FTC cells (Figure 3(d)).

CCK-8 assays were used to detect cell viability to determine the main form of curcumin-induced cell death. FTC cells were either pretreated with ferroptosis inhibitor (Fer-1 or DFO), autophagy inhibitor (CQ or 3MA), and apoptosis inhibitor (Z-VAD-FMK) for 2 h and then treated with curcumin for 24 h or subjected to curcumin treatment alone (Figures 3(e) and 3(f)) **(**Supplementary Figure 1**)**. The results indicated that the viability of FTC-133 and FTC-238 cells treated with 20 *μ*M curcumin was obviously elevated in the presence of ferrostatin-1. Moreover, at a concentration of 10 *μ*M curcumin, cell viability was significantly affected by several of the pharmacological inhibitors. We hypothesized that ferroptosis is the main cause of cell death after treatment with 20 *μ*M curcumin. Therefore, 20 *μ*M curcumin was used in the subsequent experiments.

### 3.4. Curcumin Promotes HO-1 Expression and Activates Ferroptosis in FTC Cells

**Figure 4:**
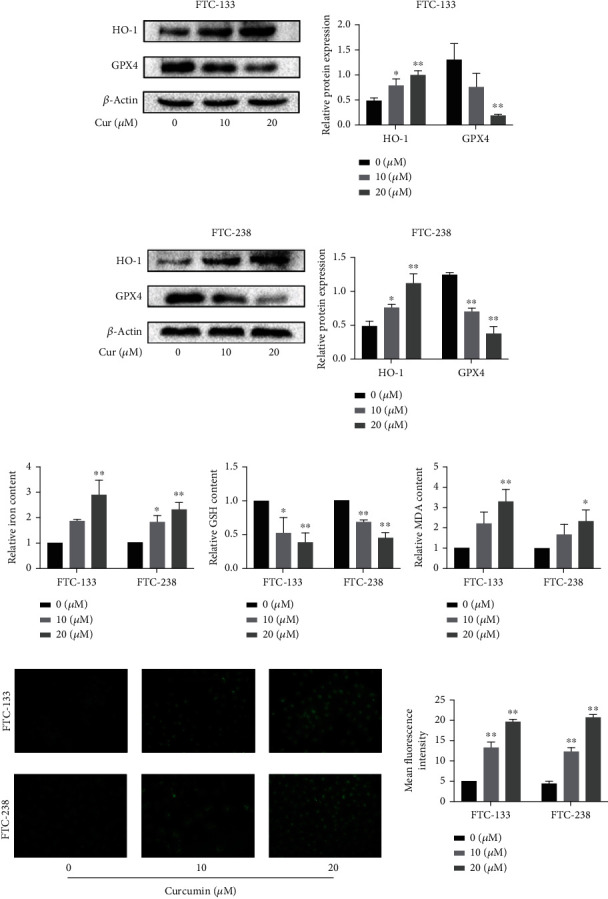
Curcumin induces ferroptosis in FTC cells. Curcumin upregulates the expression of HO-1 in FTC cells and triggers ferroptosis. (a, b) Protein expression of HO-1 and GPX4 in FTC cells after treatment with 0, 10, or 20 *μ*M curcumin. *β*-Actin was used as an internal control. (c–e) Relative iron, GSH, and MDA contents in FTC cells after treatment with 0, 10, or 20 *μ*M curcumin. (f) Determination of lipid ROS levels *via* fluorescence intensity measurements after treatment with 0, 10, or 20 *μ*M curcumin; scale bars: 50 *μ*m. ^∗^*p* < 0.05 and ^∗∗^*p* < 0.01 compared with the 0 *μ*M curcumin group. The data are presented as the mean ± standard error of the mean (SEM), *n* = 3. Statistical significance was determined by one-way ANOVA.

Although the findings indicated that curcumin-induced FTC cell death may be closely associated with ferroptosis, whether curcumin directly induces ferroptosis in FTC cells remained unclear. To investigate this possibility, FTC cells were treated with 0, 10, or 20 *μ*M curcumin for 24 h, and the levels of indicators associated with ferroptosis were determined. The results showed that curcumin decreased GPX4 protein expression and increased HO-1 protein expression (Figures 4(a) and 4(b)). In addition, curcumin increased iron content in both FTC-133 and FTC-238 cells in a dose-dependent manner (Figure 4(c)); MDA content also markedly increased with curcumin treatment (Figure 4(e)). However, the GSH contents were significantly decreased (Figure 4(d)). Furthermore, lipid ROS experiments showed that curcumin induced lipid peroxidation (Figure 4(f)). Thus, we inferred that curcumin can induce ferroptosis in FTC cells.

### 3.5. HO-1 Affects Curcumin-Induced Ferroptosis in FTC Cells by Regulating GPX4

**Figure 5:**
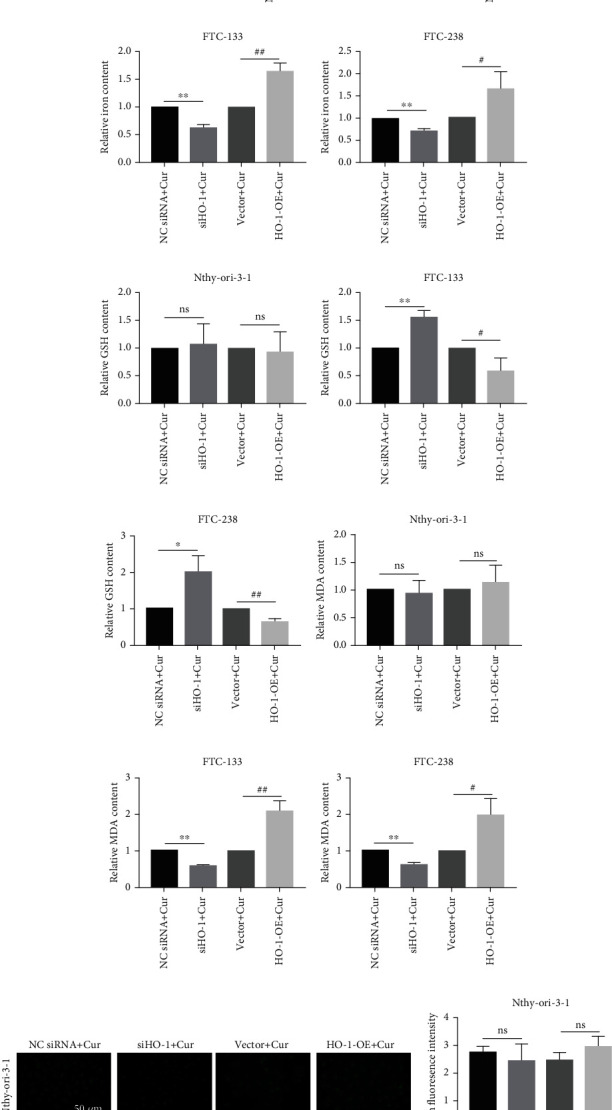
Nthy-ori-3-1 and FTC cells transfected with siHO-1/HO-1-OE and then subjected to curcumin treatment. (a–c) The relative GPX4 expression in Nthy-ori-3-1 and FTC cells transfected with siHO-1/HO-1-OE under 20 *μ*M curcumin treatment. *β*-Actin was used as an internal control. Relative iron (d–f), GSH (g–i), and MDA (j–l) contents in Nthy-ori-3-1 and FTC cells transfected with siHO-1/HO-1-OE under 20 *μ*M curcumin treatment. (m–o) Determination of lipid ROS levels *via* fluorescence intensity measurements in Nthy-ori-3-1 and FTC cells transfected with siHO-1/HO-1-OE after treatment with 20 *μ*M curcumin; scale bars: 50 *μ*m. ns: no significant difference compared with the Nc siRNA/vector + curcumin group. ^∗^*p* < 0.05 and ^∗∗^*p* < 0.01 compared with the Nc siRNA + curcumin group. ^#^*p* < 0.05 and ^##^*p* < 0.01 compared with the vector + curcumin group. The data are presented as the mean ± standard error of the mean (SEM), *n* = 3. The *t* test was used to determine statistical significance.

GPX4 is an important suppressor of ferroptosis. The regulatory relationship of GPX4 and HO-1 remains unclear. Our experimental study showed that knockdown of HO-1 promoted the GPX4 expression in curcumin-treated Nthy-ori-3-1 and FTC cells. However, the GPX4 expression was obviously inhibited when HO-1 was overexpressed (Figures 5(a)–5(c)). We hypothesized that HO-1 regulates the GPX4 expression in curcumin-induced ferroptosis. Next, we explored the role of HO-1; the transfection efficiencies of siHO-1 and HO-1-OE are shown in Figures 2(a)–2(c). HO-1 knockdown decreased the sensitivity of FTC cells to curcumin, and FTC cells showed decreased iron and MDA contents and lipid ROS levels and increased GSH contents. These results indicated that HO-1 may negatively affect the GPX4 expression in FTC cells to increase their resistance to curcumin. The HO-1 overexpression induced the opposite effects in FTC cells. However, these phenomena were not observed in Nthy-ori-3-1 cells, even when the GPX4 expression was changed (Figures 5(d)–5(o)). We concluded that curcumin-induced ferroptosis was affected by HO-1 through the regulation of GPX4 in FTC cells.

### 3.6. HO-1 Interacts with GPX4 Protein and Activates Ferroptosis

**Figure 6:**
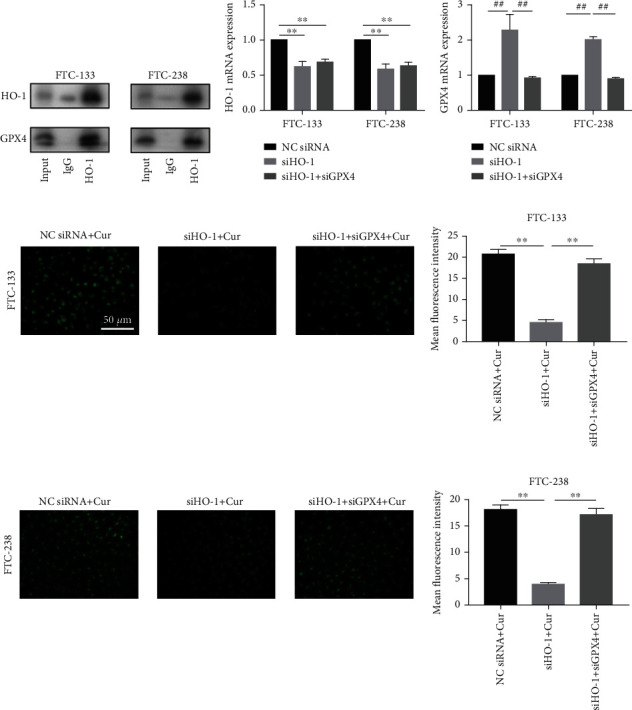
HO-1 protein interacts with GPX4 protein and promotes ferroptosis in FTC cells. (a) The interaction between HO-1 and GPX4 was confirmed by Co-IP assays, followed by western blotting. (b, c) Relative HO-1/GPX4 mRNA expression in FTC cells transfected with siHO-1 or siHO-1 + siGPX4. The expression levels of mRNA were normalized to the level of *β*-actin. ^∗∗^*p* < 0.01 compared with the Nc siRNA group. ^##^*p* < 0.01 compared with the siHO-1 group. (d, e) Determination of lipid ROS levels *via* fluorescence intensity measurements in FTC cells transfected with siHO-1 or siHO-1 + siGPX4 after treatment with 20 *μ*M curcumin; scale bars: 50 *μ*m. ^∗∗^*p* < 0.01 compared with the siHO-1 + curcumin group. The data are presented as the mean ± standard error of the mean (SEM), *n* = 3. One-way ANOVA (b–e) was used to determine statistical significance.

Co-IP experiments were performed in FTC cells to confirm that HO-1 can specifically interact with GPX4 and regulate its expression (Figure 6(a)). To confirm that HO-1 induces ferroptosis by negatively regulating GPX4, FTC cells were cotransfected with siHO-1 and siGPX4 (Figures 6(b) and 6(c)). As shown in Figure 6(c), when FTC cells were transfected with siHO-1, the GPX4 mRNA expression was obviously increased. Subsequently, lipid ROS experiments were used to test the extent to which ferroptosis occurs. The results showed that transfection with siGPX4 reversed the inhibitory effects of siHO-1 on curcumin-induced ferroptosis and led to an increase in lipid ROS levels (Figures 6(d) and 6(e)).

## 4. Discussion

FTC is an extremely aggressive malignant cancer of the thyroid and is more aggressive than PTC [25]. In this study, we aimed to identify a molecular mechanism underlying FTC and to develop corresponding interventions. We obtained three main findings: (1) HO-1 is abnormally highly expressed in FTC tissues compared with adjacent tissues. (2) The HO-1 overexpression promotes cell death, and these effects may be induced by activating ferroptosis. (3) Curcumin suppresses the tumorigenesis of FTC by activating HO-1, potentially *via* activation of the ferroptosis signalling pathway. By catabolizing toxic intracellular haem, HO-1 exerts antioxidant, antiapoptotic, and cytoprotective effects. The catabolites of haem degradation can facilitate the angiogenesis and metastasis of cancer cells, as well as their evasion of immune surveillance, thereby influencing cellular functionality and promoting tumor progression [26]; these processes may explain the higher HO-1 expression in the more invasive FTC-238 cells than in the less invasive FTC-133 cells. However, a growing number of studies have shown that HO-1 plays a dominant role in ferroptosis [27–30]. Experiments with cells confirmed that the HO-1 overexpression obviously inhibited cell viability under erastin treatment; this inhibition may have been related to the initiation of ferroptosis.

Several early studies indicated that ferroptosis is not associated with autophagy. In contrast, recent research suggests that autophagy promotes ferroptosis by selectively degrading antiferroptosis regulators [31]. A growing body of evidence demonstrates that excessive autophagy and lysosome activity can facilitate ferroptosis by accumulating iron or lipid ROS [32]. However, the mechanism of autophagy-dependent ferroptosis remains relatively poorly understood. Curcumin mediates the intricate crosstalk among autophagy, apoptosis, and ferroptosis due to its antioxidant, anti-inflammatory, and antiproliferative properties [33, 34]. Furthermore, curcumin has been demonstrated to suppress FTC cells, and in the present study, ferrostatin-1 obviously reversed the inhibitory effects of 20 *μ*M curcumin on FTC cells, but it is still unclear whether curcumin suppresses FTC cells by triggering ferroptosis.

Ferroptosis is a type of iron-dependent oxidative cell death [35] and is characterized by high iron contents, high MDA levels [36], and GSH depletion [37]. In addition, ferroptosis is induced by the inactivation of GPX4, which results in the accumulation of lipid ROS. In this study, we demonstrated that HO-1 exerted its function in FTC cells by regulating ferroptosis under curcumin treatment. However, HO-1 actually plays a dual role in cancer cells. Under normal physiological activation, HO-1 scavenged ROS and demonstrates cytoprotective effects, while excessive activation of HO-1 causes ferroptosis by increasing ROS levels and iron concentrations [6]. GPX4, a monomeric glutathione peroxidase, can decrease hydroperoxide levels in complex lipids [38]. The key event of ferroptosis is the enhancement of lipid peroxidation induced by GPX4 inhibition [39]. In addition, GPX4 has been linked to multiple cancers because of its cancerous effects [40]. Interestingly, there was a negative regulatory relationship between HO-1 and GPX4 in the present study. Knockdown of GPX4 reversed siHO-1-mediated inhibition of curcumin-induced ferroptosis. The above experimental results demonstrate that knockdown of HO-1 inhibits ferroptosis by upregulating the GPX4 expression in FTC cells.

## 5. Conclusion

We conclude that curcumin inhibits the tumorigenesis of FTC *via* HO-1-induced activation of the ferroptosis signalling pathway. Further research is needed to determine the specific mechanism underlying the effect of curcumin on FTC; however, for patients who cannot tolerate surgery or are difficult to diagnose, it may be possible to develop a therapeutic pathway towards the remission of FTC based on our findings.

## Figures and Tables

**Table 1:** Sequences of siRNAs for HO-1 and GPX4.

Gene	Sequence (5′–3′)
HO-1 siRNA sense	GGGUGAUAGAAGAGGCCAATT
HO-1 siRNA antisense	UUGGCCUCUUCUAUCACCCTT
GPX4 siRNA sense	CAGGGAGUAACGAAGAGAUTT
GPX4 siRNA antisense	AUCUCUUCGUUACUCCCUGTT

**Table 2:** Primers used for real-time PCR.

Gene	Sequence (5′–3′)
HO-1 forward	ACACCCAGGCAGAGAATGCT
HO-1 reverse	CGAAGACTGGGCTCTCCTTGT
GPX4 forward	ATCGACGGGCACATGGTTAA
GPX4 reverse	CAGGATCCGCAAACCACACT
*β*-Actin forward	GGCACCCAGCACAATGAA
*β*-Actin reverse	TAGAAGCATTTGCGGTGG

**Table 3:** Immunohistochemistry analysis.

	Total	Positive	Negative	*χ* ^2^	*p* value
Cancer	33	20	13	4.93	0.026^∗^
Normal	33	11	22

^∗^ *p* < 0.05.

## Data Availability

The data used to support the findings of this study are included within the article.

## Supplementary Materials

Supplementary Materials Supplementary Figure 1: effects of various inhibitors on FTC cells under curcumin treatment.
