# Supplementary material for: Curcumin Induces Ferroptosis in Follicular Thyroid Cancer by Upregulating HO-1 Expression
Source: Oxid Med Cell Longev. 2023 Jan 14;2023:6896790. doi: 10.1155/2023/6896790 (PMC9867595; doi:10.1155/2023/6896790)
Supplement: Supplementary Materials — Supplementary Figure 1: effects of various inhibitors on FTC cells under curcumin treatment. [file 6896790.f1.docx]

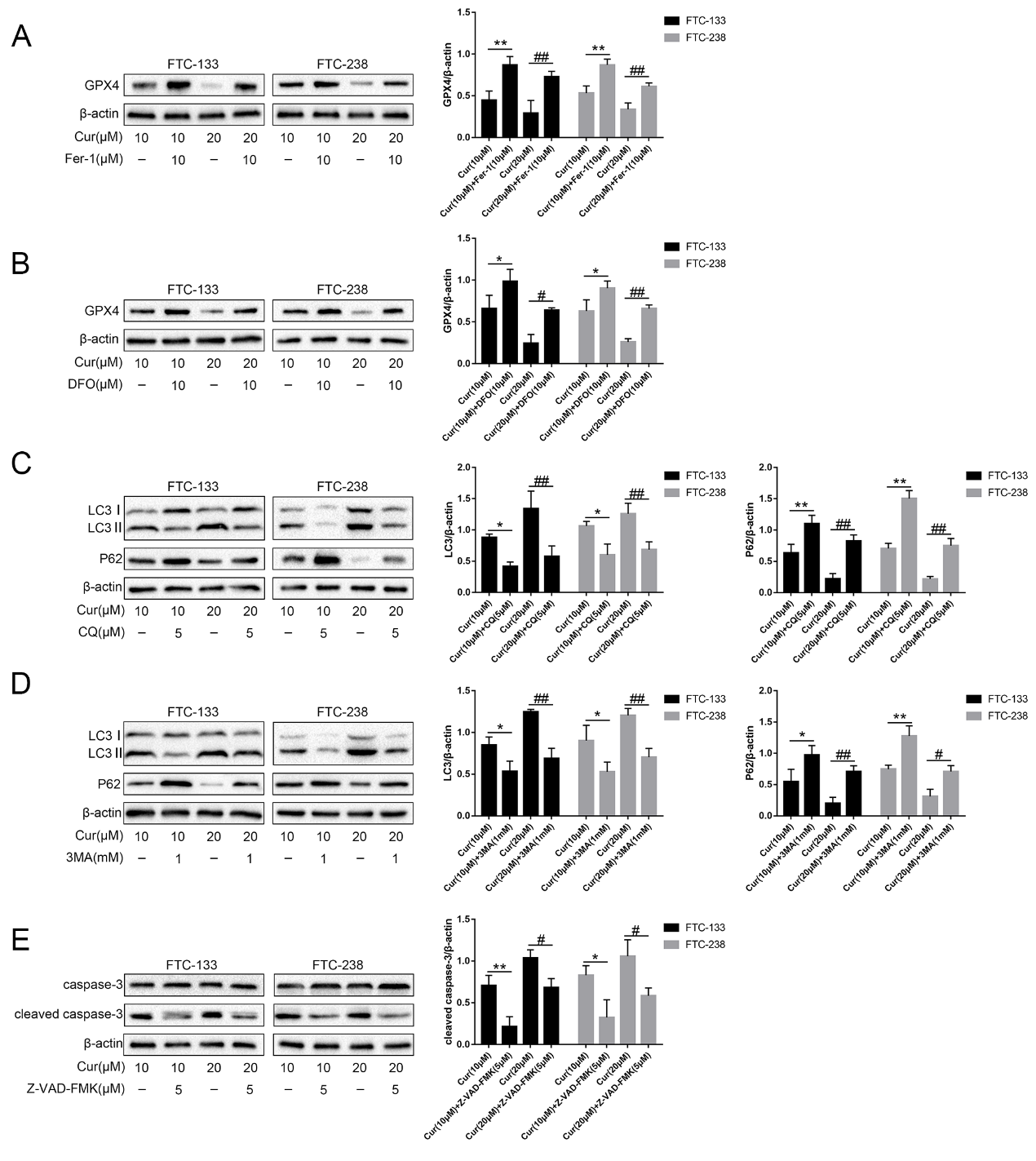


**Supplementary Fig. 1 Effects of various inhibitors on FTC cells under curcumin treatment**

Protein expression of GPX4 in the presence of Fer-1 (A) and DFO (B). Protein expression of P62 and LC3 in the presence of CQ (C) and 3MA (D). Protein expression of caspase-3 and cleaved caspase-3 in the presence of Z-VAD-FMK (E). β-actin was used as an internal control. **p*<0.05 and ***p*<0.01 compared with 10 μM curcumin alone. ^#^*p*<0.05 and ^##^*p*<0.01 compared with 20 μM curcumin alone. The data are presented as the mean ± standard error of the mean (SEM), *n*=3. The *t* test was used to determine statistical significance.
